# Effects of Mediterranean Diet, DASH Diet, and Plant-Based Diet on Outcomes among End Stage Kidney Disease Patients: A Systematic Review and Meta-Analysis

**DOI:** 10.3390/clinpract13010004

**Published:** 2022-12-28

**Authors:** Mariam Charkviani, Charat Thongprayoon, Supawit Tangpanithandee, Pajaree Krisanapan, Jing Miao, Michael A. Mao, Wisit Cheungpasitporn

**Affiliations:** 1 Division of Nephrology and Hypertension, Mayo Clinic, Rochester, MN 55905, USA; 2 Chakri Naruebodindra Medical Institute, Faculty of Medicine Ramathibodi Hospital, Mahidol University, Samut Prakan 10540, Thailand; 3 Division of Nephrology, Department of Internal Medicine, Faculty of Medicine, Thammasat University, Pathum Thani 12120, Thailand; 4 Department of Nephrology and Hypertension, Mayo Clinic, Jacksonville, FL 32224, USA

**Keywords:** end stage kidney disease, hemodialysis, peritoneal dialysis, DASH, Mediterranean diet, plant-based diet

## Abstract

Background: The Mediterranean, Dietary Approach to Stop Hypertension (DASH), and plant-based diets may provide cardiovascular benefit to the general population. However, data on their effect on end stage kidney disease (ESKD) patients are limited. This systematic review aims to assess the impact of Mediterranean, DASH, and plant-based diets on outcomes among ESKD patients. Methods: A literature review was conducted in EMBASE, MEDLINE, and Cochrane databases from inception through September 2022 to identify studies that assess the clinical outcomes of Mediterranean, DASH, or plant-based diets on ESKD patients on hemodialysis (HD) or peritoneal dialysis (PD). Effect estimates from the individual studies were derived utilizing the random-effect, generic inverse variance approach of DerSimonian and Laird. Results: Seven studies with 9400 ESKD patients (8395 HD and 1005 PD) met the eligibility criteria and were included in the data analysis. Pooled odds ratios (ORs) of mortality for ESKD patients who adhered to the Mediterranean versus plant-based diet were 0.49 (95% CI: 0.07–3.54; two studies, I^2^ = 67%) and 0.87 (95% CI: 0.75–1.01; two studies, I^2^ = 0%), respectively. Data on mortality for ESKD patients on a DASH diet were limited to one study with an OR of 1.00 (95% CI: 0.89–1.12). The pooled OR of cardiovascular mortality among ESKD patients who adhered to a plant-based diet was 0.86 (95% CI: 0.68–1.08; two studies, I^2^ = 0%), compared to those who did not. Data on cardiovascular mortality among those with Mediterranean and DASH diet were limited to one study with ORs of 1.14 (95% CI: 0.90–1.43) and 1.19 (95% CI: 0.99–1.43), respectively. Mediterranean diet adherence was found to be associated with reduced risk of left ventricular hypertrophy (LVH) with an OR of 0.82 (95% CI: 0.68–0.99) in a study including 127 ESKD patients. The risk of hyperkalemia was not significant among those with a plant-based diet with an OR of 1.00 (95% CI: 0.94–1.07) in a study including 150 ESKD patients. Conclusions: While our systematic review demonstrated no significant associations of Mediterranean, DASH, and plant-based diets with reduced all-cause mortality or cardiovascular mortality, there was also no evidence that suggested harmful effects of these diets to ESKD patients.

## 1. Introduction

Patients with end stage kidney disease (ESKD) on hemodialysis (HD) have an increased risk of premature mortality that is approximately ten times higher than in the general population [1], and a 40% increased risk of death is from cardiovascular disease [2]. There is limited evidence on whether any preventive or treatment strategies significantly reduce the risk of mortality in this high-risk vulnerable population. Lifestyle and dietary changes are potentially modifiable factors that have been shown to have significant public health benefits in the general population. Studies have also shown that a plant-based diet is associated with lower risk of cardiovascular morbidity and mortality in the general population [3,4,5]. The Dietary Approach to Stop Hypertension diet (DASH) and the Mediterranean diet emphasize increased fruits, vegetables, and fish intake, and recommend reducing fat, saturated fat, refined sugars and meat. Studies have shown that these diets are associated with a 10–30% reduction in cardiovascular event mortality in the general population [6,7,8]. This effect is thought to be achieved by the reduction of insulin resistance, oxidative stress, and inflammation, and improved serum lipids, blood pressure (BP), endothelial function, and arterial compliance [7,9,10].

Mediterranean diet adherence is shown to be associated with reduced mortality among patients with chronic kidney disease (CKD) [11] and evidence also suggests that it may prevent or decrease progression of CKD [12]. A recent systematic review also showed that each one-point-higher adherence to the Mediterranean diet was associated with a 10% reduction of progression of CKD [13]. However, the effects of the DASH diet on clinical outcomes among ESKD patients are inconsistent, and it remains unclear if it has any beneficial effect in this population [14,15,16,17,18,19,20]. Current dietary guidelines for ESKD patients usually recommend the restriction of specific nutrients (sodium, potassium, phosphorus, etc.) and recommend high protein and calorie intake [21,22,23]. However, there is limited evidence showing the clinical benefits of restricted nutrients in ESKD patients. The literature has been inconsistent regarding the benefits of a DASH diet in the chronic kidney disease population, and only a small number of studies are available in ESKD patients [14,15,16,17,18,19,20]. In this systematic review, we evaluated the impact of a DASH, Mediterranean, or plant-based diet on clinical outcomes, including all-cause and cardiovascular mortality, left ventricular hypertrophy (LVH), and hyperkalemia in ESKD patients.

## 2. Materials and Methods

### 2.1. Information Sources and Search Strategy

The protocol for this systematic review is registered with PROSPERO (international Prospective Register of Systematic Reviews; No: CRD42022356071). A systematic literature search was performed using the Ovid Medline, EMBASE, and Cochrane databases from inception through September 2022 to identify all original studies that assessed the incidence of all-cause and cardiovascular mortality, LVH, and hyperkalemia in ESKD patients treated on Mediterranean, DASH or plant-based diets. The systematic literature review was individually conducted by two investigators (M.C. and C.T.) using the search strategy as described in online Supplementary Data S1. A manual search for additional potentially relevant studies utilizing references of the included articles was also conducted. No language limitation was applied. Any conflicting decisions were resolved by mutual consensus. This study was performed in agreement with the PRISMA [24] (Preferred Reporting Items for Systematic Reviews and Meta-Analysis) Statement.

### 2.2. Selection Criteria

Eligible studies included case-control, cross-sectional, or cohort studies that evaluated the incidence of cardiovascular and all-cause mortality, LVH, and hyperkalemia in ESKD patients that were on Mediterranean, DASH, or plant-based diets. Studies had to provide the estimated incidence or effect estimates (odds ratios (OR), relative risk (RR), or hazard ratios (HR)) with 95% confidence intervals (CI). Inclusion was not restricted by study size. The quality of each study was evaluated by investigators using the validated Newcastle–Ottawa quality assessment scale [25] for case-control and cohort studies and the modified Newcastle–Ottawa scale [26] for cross-sectional studies.

### 2.3. Data Abstraction

A structured data collection report was adopted to derive the following information from included studies: study title, first author name, publication year, year of the study, country where the study was conducted, number of participants, participant characteristics, definition of diet including Mediterranean, DASH or plant-based diet, definition of ESKD, and incidence of all-cause mortality, cardiovascular mortality, LVH, and hyperkalemia with associated adjusted effect estimates with 95% CI. To ensure precision, the data extraction process was independently performed by three investigators.

### 2.4. Statistical Analysis

Data analysis was performed using the Comprehensive Meta-analysis tool. Adjusted point estimates from each study were assembled by the generic inverse variance method of DerSimonian and Laird [27], which provided the weight of each study in the pooled analysis based on its variance. Cochran’s Q test and I^2^ statistics were used to determine the between-study heterogeneity; 51–75% represents moderate heterogeneity, and more than 75% represents high heterogeneity [28]. An eager regression symmetry test was used to assess for publication bias [29]. A *p* value < 0.05 was considered statistically significant for all analyses.

## 3. Results

**Figure 1:**
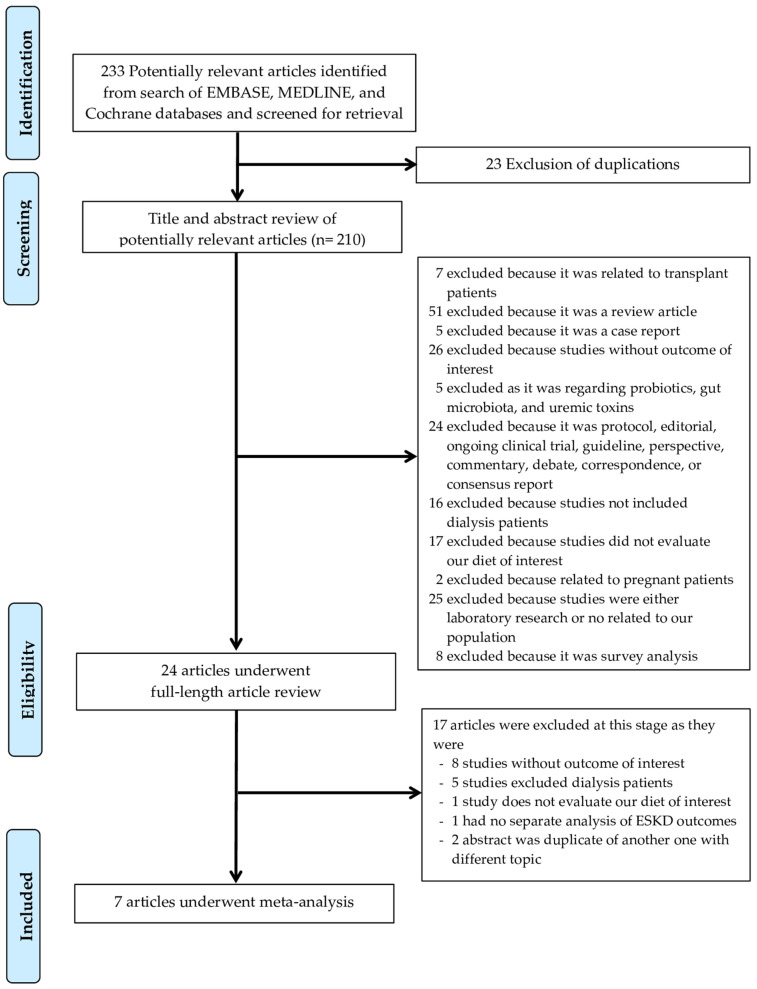
Literature Review Process.

A total of 233 potentially eligible articles were identified using our search strategy. After the exclusion of 209 articles (because they were case reports, duplicates, correspondence, review articles, in vitro studies, or animal studies), this left 24 articles for full-length review. Seventeen studies were further excluded as they either did not report outcomes of interest, excluded dialysis patients, did not evaluate the diet of interest, or had no separate analysis of ESKD outcomes. The final analysis included seven articles [14,15,16,17,18,19,20], consisting of 9400 ESKD patients, from which 8395 were HD patients and 1005 were peritoneal dialysis (PD) patients. The literature retrieval, review, and selection process are demonstrated in Figure 1. The characteristics and quality assessment of the included studies are presented in Table 1.

### 3.1. Incidence of All-Cause and Cardiovascular Mortality in ESKD Patients on DASH, Mediterranean, or Plant-Based Diet

**Figure 2:**
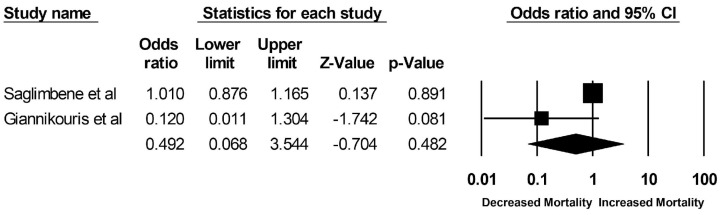
Forest plots of the included studies assessing outcomes defined as DASH and Mediterranean and all-cause mortality [15,20].

**Figure 3:**
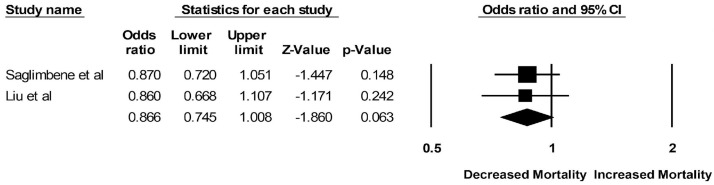
Forest plots of the included studies assessing outcomes defined as a plant-based diet and all-cause mortality [17,18].

**Figure 4:**
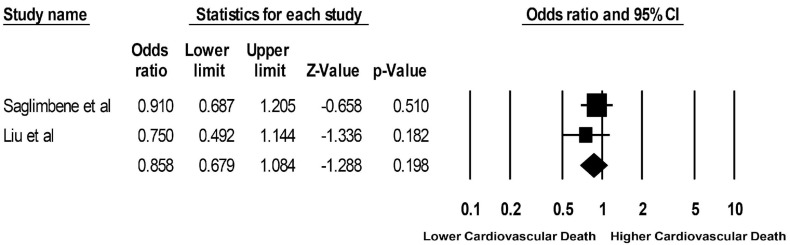
Forest plots of the included studies assessing outcomes defined as a plant-based diet and cardiovascular mortality [17,18].

Pooled odds ratios (ORs) of mortality among ESKD patients who adhered to a Mediterranean diet versus plant-based diet were 0.49 (95% CI: 0.07–3.54; two studies, I^2^ = 67%) and 0.87 (95% CI: 0.75–1.01; two studies, I^2^ = 0%), respectively (Figure 2 and Figure 3). Mortality data for ESKD patients on a DASH diet were limited to one study with an OR of 1.00 (95% CI: 0.89–1.12). The pooled OR of cardiovascular mortality among ESKD patients who adhered to a plant-based diet was 0.86 (95% CI: 0.68–1.08; two studies, I^2^ = 0%), compared to those who did not (Figure 4). Data on cardiovascular mortality in ESKD patients on a Mediterranean diet and DASH diet were limited to one study with ORs of 1.14 (95% CI: 0.90–1.43) and 1.19 (95% CI: 0.99–1.43), respectively.

### 3.2. Incidence of LVH and Hyperkalemia Related to DASH, Mediterranean, and Plant-Based Diet

Mediterranean diet adherence was found to be associated with a reduced risk of LVH with an OR of 0.82 (95% CI: 0.68–0.99) in a study including 127 ESKD patients [14]. The risk of hyperkalemia was not significantly increased in ESKD patients with a plant-based diet with an OR of 1.00 (95% CI: 0.94–1.07) in a study including 150 ESKD patients [19]. A funnel plot was not drawn because of the limited number of studies. As a rule of thumb, tests for funnel plot asymmetry should be utilized only when there are ≥10 study groups. Due to the limited number, the power of the test is too low to distinguish chance from real asymmetry [30].

## 4. Discussion

This systematic review provides new insight into the effect of dietary patterns on clinical outcomes in ESKD patients. Current dietary recommendations mainly focus on the role of individual nutrients rather than whole dietary patterns. These current recommendations include a diet low in sodium, potassium, and phosphorus, and high in energy and proteins [23]. In contrast, the Mediterranean, DASH, and plant-based diets, which have been shown to improve all-cause and cardiovascular mortality in the general population [3,4,5,6,7,8], are rich in potassium and phosphorus while low in animal proteins. There is limited evidence regarding the impact of these dietary patterns on ESKD patients. In this systematic review involving 9400 patients with ESKD on dialysis (both HD and PD), we demonstrated that there was no significant impact of dietary pattern (Mediterranean, DASH, or plant-based) on overall incidence of all-cause or cardiovascular mortality in ESKD patients. However, we also showed that despite the lack of benefits on mortality outcomes, these dietary patterns did not contribute to harm in ESKD patients with respect to hyperkalemia, mortality, or adverse cardiovascular outcomes.

The lack of benefit between these dietary patterns and mortality can have several explanations. The magnitude of ESKD’s deleterious effect on health in a unique population with multiple comorbidities compared to the general population may be so significant that it can override any discernible beneficial impact of these dietary patterns [31,32,33,34,35]. The higher frailty and lower socioeconomic status in the ESKD population compared to the general population could also have biased the results [36,37,38,39]. Finally, the follow-up duration for these included studies was relatively short, so it may have reduced the detection of any significant association.

The absence of a cardiovascular benefit from a Mediterranean, DASH, or plant-based diet can be hypothesized that in the general population, the cardioprotective mechanisms of these diets are largely driven by improved lipid, glycemic, and blood pressure control [3,4,5,40,41]. In HD patients, it has been shown that there are multiple nontraditional factors that can be associated with increased cardiovascular events and mortality, including increased oxidative stress, inflammation, uremic effects, altered calcium-phosphate regulation, and endothelial dysfunction [22,42]. That could explain why any beneficial physiological changes caused by a Mediterranean, DASH, or plant-based diet may not be sufficient to improve cardiovascular outcomes.

Higher adherence to a Mediterranean diet was associated with decreased adverse cardiovascular outcome markers as measured by LVH in ESKD patients [16]. Becharaki et al. [16] also showed that a higher Mediterranean diet score was associated with a lower prevalence of crescentic and eccentric cardiac geometry. A prior meta-analysis that evaluated secondary cardiovascular outcomes for a Mediterranean diet in the general population did not show a significant association [43], however, they did not evaluate the geometry of the heart. There is limited literature regarding the dietary effect on cardiac geometry in CKD patients, but it has been shown that CKD was associated with significant changes in left ventricular geometry [44,45]. Previous studies have demonstrated that diets rich in phosphorus and sodium are associated with increased cardiac hypertrophy in ESKD patients [46,47]. A Mediterranean diet with lower salt and phosphorus compared to the Western diet could explain the reduced prevalence of LVH in ESKD patients. However, there might be some unattributed dietary component in the Mediterranean diet translating to improved cardiovascular outcomes [15].

Our systematic review interestingly showed that despite being on a Mediterranean, DASH, or plant-based diet that is rich in potassium, ESKD patients did not develop hyperkalemia compared to controls. Notably, while our study did not show significant the benefit of a Mediterranean, DASH, or plant-based diet on ESKD patients for the respective outcomes noted above, it also, conversely, did not show harm with these dietary patterns. This review may provide evidence against the commonly accepted blanket practice of dietary modifications in ESKD patients that includes the avoidance of many plant-based diets due to the concern of hyperkalemia.

Efforts to mitigate the significant morbidity and mortality in ESKD patients have been largely frustratingly inadequate. Future studies are needed that evaluate the efficacy of a multitarget approach that encompasses a simultaneous treatment strategy that revisits prior treatment efforts such as dietary therapy, statin, blood pressure control, fluid management, CKD-MBD, and other interventions. As ESKD is a solitary organ system failure that results in systemic and widespread multiorgan deleterious effects, a preventative and therapeutic approach may require a similar comprehensive treatment strategy in this high-risk population.

This study has several limitations. First, there was low level but statistically significant heterogeneity between studies in this meta-analysis. The possible source of this heterogeneity includes the difference in assessment of the dietary patterns and comparator groups utilized among the included studies. However, this heterogeneity was relatively insignificant in the analyses assessing the incidence of all-cause mortality, cardiovascular mortality, and rate of hyperkalemia. Second, this is a meta-analysis of observational studies. Thus, it can only at best identify associations between dietary patterns and adverse outcomes in ESKD, but not a causal relationship. Another limitation is that this meta-analysis did not investigate other cardiovascular outcomes of interest (such as LVEF, diastolic dysfunction, NYHA class, history of PCI or CABG) or other adverse effects (nutritional status such as low BMI, exercise tolerance, and serum albumin). In addition, while this current study is the first systematic review and meta-analysis that summarizes all available data on Mediterranean, DASH, or plant-based diets in ESKD patients, the number of relevant studies existing and included in the meta-analysis are limited, and thus future studies with long-term follow-up are required.

## 5. Conclusions

While our systematic review demonstrated no significant beneficial associations of a Mediterranean, DASH, or plant-based diet with reduced all-cause mortality or cardiovascular mortality, there was also no evidence suggesting the harmful effect of mortality or hyperkalemia of these diets on ESKD patients. While Mediterranean diet adherence was found to be associated with a reduced risk of LVH among ESKD patients, future large prospective multi-center studies are still required.

## Figures and Tables

**Table 1:** Characteristics of included studies.

Study	Saglimbene et al. [15]	Saglimbene et al. [18]	Bacharaki et al. [16]	Gonzalez-Ortiz et al. [19]	Liu et al. [17]	Giannikouris et al. [20]	**Bacharaki et al. [14]**
Year	2018	2020	2022	2021	2020	2019	2017
Study design	Cohort study	Cohort Study	Cross-sectional	Cohort study	Cohort study	Cohort study	Cross-sectional
Number of Patients	8110 HD patients	8110 HD patients	127 patients overall, 69 on HD, 58 on PD	150 HD patients	884 PD patients	129 patients overall, 66 on HD, 63 on PD	53 PD patients
Age	mean age 63.1 ± 15	mean age 63 ± 15	mean age 62 ± 15	mean age 42 ± 18	mean age 57.7 ± 14.8	mean age 62 ± 15	mean age 62 ± 14
Sex	58% male	58% male	54% male	41% male	49% male	60% male	51% male
Type of Diet	Mediterranean diet DASH diet	Plant-Based diet	Mediterranean diet	Plant-Based diet	Plant-based protein—total protein ratio	Mediterranean diet	Mediterranean diet
Follow Up Time	2.7 years	2.7 years	N/A	1 year	3.8 years	1.7 years	N/A
All-Cause Mortality OR (95% CI)	Mediterranean diet Q3 vs. Q1 1.01 (0.88–1.17) DASH diet Q3 vs. Q1 1.00 (0.89–1.12)	Q4 vs. Q1 0.87 (0.72–1.05)	N/A	N/A	Q3 vs. Q1 0.86 (0.67–1.11)	High vs. low MDS group 0.12 (0.01–0.96)	N/A
Cardiovascular mortality OR (95% CI)	Mediterranean diet Q3 vs. Q1 1.14 (0.90–1.43) DASH diet Q3 vs. Q1 1.19 (0.99–1.43)	Q4 vs. Q1 0.91 (0.69–1.21)	N/A	N/A	Q3 vs. Q1 0.75 (0.49–1.14)	N/A	N/A
LVH OR (95% CI)	N/A	N/A	Per 1 point 0.82 (0.68–0.99)	N/A	N/A	N/A	Per 1 point 0.75 (0.57–0.93)
Hyperkalemia OR (95% CI)				Per 1 unit 1.00 (0.94–1.07)			
Confounder adjustment	Random effect to estimate the associations between dietary pattern scores and mortality, sex, daily physical activity, education, diabetes, smoking, MI, vascular access type, BMI, albumin, Charlson comorbidity index, score, age, phosphorus, calcium, Hemoglobin, Kt/V, energy intake	Random effect to estimate the associations between dietary pattern scores and mortality, sex, daily physical activity, education, diabetes, smoking, MI, vascular access type, BMI, albumin, Charlson comorbidity index, score, age, phosphorus, calcium, Hemoglobin, Kt/V, energy intake	Dialysis mode, sex, age, PVD, CAD, diabetes, BMI, plasma phosphorus, serum albumin, serum magnesium	Age, sex, dialysis vintage, occupation, diabetes, hypertension, CVD history, RAS blockers, loop diuretics use and servings of fat	Age, gender, income, Diabetes Mellitus, CVD, time averaged variables, BMI, hemoglobin, albumin, C-reactive protein, plasma protein level, parathyroid hormone, total energy intake, total fiber intake	Age, cardiovascular disease, serum albumin and malnutrition- inflammation score	N/A
Quality assessment	S4, C1, O3	S4, C1, O3,	S2, C1, O3	S4, C1, O2	S3, C1, O3	S4, C1, O3	S2, C0, O3

HD, hemodialysis; PD, peritoneal dialysis; CAD Coronary artery disease, PVD; peripheral vascular disease; RAS, renin angiotensin system; BMI, body mass index; MDS, Mediterranean adherence diet score; N/A, not available. S, selection; C, comparability; O, outcome; Q, interquartile range.

## Data Availability

The data that support the findings of this study are available on request from the corresponding author (W.C.).

## Supplementary Materials

The following supporting information can be downloaded at: https://www.mdpi.com/article/10.3390/clinpract13010004/s1.
