# Supplementary material for: Effects of Mediterranean Diet, DASH Diet, and Plant-Based Diet on Outcomes among End Stage Kidney Disease Patients: A Systematic Review and Meta-Analysis
Source: Clin Pract. 2022 Dec 28;13(1):41–51. doi: 10.3390/clinpract13010004 (PMC9844348; doi:10.3390/clinpract13010004)
Supplement: Supplementary file 1 [file clinpract-13-00004-s001.zip › clinpract-2077966-supplementary.pdf]

Database: Embase <1988 to 2022 Week 33>, Ovid MEDLINE(R) <1946 to September 2022>, EBM Reviews - Cochrane Central Register of Controlled Trials < September 2022>, EBM Reviews - Cochrane Database of Systematic Reviews <2005 to September, 2022>
Search Strategy:
----
1 plant based$.mp.
2 Mediterranean diet$.mp.
3 exp mediterranean diet$/
4 Mediterranean food$.mp.
5 Dash diet$.mp.
6 exp DASH diet$/
7 Dietary approach to stop hypertension$.mp.
8 exp Dietary approach to stop hypertension$/
9 Dialysis$.mp.
10 exp dialysis$/
11 hemodialysis$.mp.
12 exp hemodialysis$/
13 peritoneal dialysis$.mp.
14 exp peritoneal dialysis$/
15 9 or 10 or 11 or 12 or 13 or 14
16 1 and 15
17 1 or 2 or 3 or 4 or 5 or 6 or 7 or 8
18 15 and 17
